# A Comparative Evaluation of Contemporary Aesthetic Perceptions Between Dental Students and Laypersons

**DOI:** 10.1155/ijod/7428492

**Published:** 2026-10-09

**Authors:** Beyza Betul Sencan, Sumeyye Kılıclar

**Affiliations:** ^1^ Department of Prosthodontics Faculty of Dentistry Sakarya University Sakarya Türkiye sakarya.edu.tr

**Keywords:** aesthetic, dental, layperson, perception, social media

## Abstract

**Introduction:**

Aesthetic perception of the smile plays a critical role in social interactions and facial attractiveness. With the increasing influence of social media, aesthetic standards have become more dynamic and may differ between individuals with professional dental education and laypersons. This study aimed to compare dental aesthetic perceptions between dental students and laypersons and to investigate the influence of social media.

**Methods:**

This cross‐sectional study included 242 participants aged 18–30 years, comprising 130 dental students and 112 laypersons. A 10‐item online questionnaire was used. Questions 2–6 assessed preferences for tooth colour, gingival display, lip fullness, diastema and ‘bunny teeth’. Questions 7–10 evaluated social media use and its perceived influence. Data were analysed using R software, with significance set at *p* < 0.05.

**Results:**

White tooth colour and normal alignment were preferred in both groups. Dental students showed a higher preference for reduced gingival display. No significant differences were found for lip fullness or diastema. Social media use was high(96.6%), with Instagram the most used platform (80.3%) and more influential among dental students. Most participants reported that social media affects their aesthetic perception.

**Conclusions:**

Dental students and laypersons showed largely similar aesthetic preferences, with minor differences. Social media has a notable influence on aesthetic perception in both groups.

**Clinical Significance:**

Most participants, including dental students, reported that social media influences their perception of dental aesthetics. Recognising this influence may help clinicians better understand patient expectations and improve communication in aesthetic dental practice.

## 1. Introduction

In the contemporary world, aesthetic perceptions have gained increasing prominence, becoming more dynamic and influential than in the past [1].

A smile, as one of the most distinctive elements of a facial expression, plays a central role in interpersonal communication. Individuals with more aesthetically pleasing smiles are often associated with improved career opportunities and higher levels of life satisfaction [2]. In contrast, inadequate dental aesthetics may contribute to reduced self‐confidence, negatively affecting social interactions as well as academic and professional performance [3].

Dental aesthetics as an integral component of overall facial appearance have also undergone a significant evolution in response to these changing perceptions. Individual preferences continue to diversify, while social, cultural and technological factors play a major role in shaping how dental beauty is perceived and valued across different societies [4, 5].

An ideal smile is generally influenced by several dental and facial characteristics, including tooth colour, shape, size, alignment and the presence or absence of diastema [6, 7]. The perception of these features varies considerably among individuals, depending on determinants such as age, gender, social background, ethnicity and personal aesthetic awareness [4, 8]. Previous studies have suggested that dental students often evaluate dental aesthetics with a more analytical and profession‐oriented approach, whereas individuals without formal dental education tend to assess smiles based on more general or subjective aesthetic standards [1, 5, 9–11]. Therefore, comparing these two groups provides valuable insight into whether professional training influences aesthetic judgement and how these perceptions differ from those of the general population. In this context, the widespread use of social media has significantly influenced the formation and transformation of the aesthetic perception, particularly among young individuals [12]. The idealised representations of faces and smiles presented on digital platforms have become powerful reference points that shape expectations and evaluative criteria regarding the aesthetics [13]. This process not only leads to a more defined and intensified perception of aesthetic norms but also increases awareness and sensitivity toward key facial components, such as dental aesthetics. In addition, the rapid expansion of social media has significantly reshaped aesthetic norms, particularly in relation to the dental appearance. Platforms such as Instagram, TikTok and Snapchat expose users to idealised images of smiles and cosmetic dental treatments, influencing aesthetic expectations—especially among younger populations and women—and increasing the demand for dental aesthetic procedures [14]. Given this influence, it is important to investigate whether the frequency of social media use is associated with differences in aesthetic preferences.

Given these developments, understanding how different groups perceive dental aesthetics has become increasingly important. The present study aims to evaluate and compare the aesthetic perceptions of dental students and laypersons using selected dental aesthetic parameters. In addition, the potential influence of social media usage on these perceptions is also investigated. Such analysis may provide valuable insights for dental education, enhancing students’ ability to approach future clinical decisions and treatment planning with a broader and more patient‐centred aesthetic perspective.

## 2. Materıals and Methods

This cross‐sectional study was conducted among 4th‐ and 5th‐year students of the Sakarya University Faculty of Dentistry and individuals without formal dental education (laypersons). A 10‐item online questionnaire was created by the researchers using Google Forms (Google LLC, Mountain View, CA, USA) (Table 1).

**Table 1:** Survey questions.

(1) Gender Male Female Prefer not to answer (2) Which of the following images do you find more aesthetic? Refer to Figure 1 (3) Considering the existing gingival visibility, which image do you find more aesthetic? Refer to Figure 2 (4) Considering lip fullness, which image do you find more aesthetic? Refer to Figure 3	(5) Which of the images below do you find more aesthetic? Refer to Figure 4 (6) Which of the images below do you find more aesthetic? Refer to Figure 5
(7) Do you use social media? • Yes • No (8) Which social media platform do you use most frequently? • Instagram • TikTok • X • Facebook • Pinterest • Snapchat (9) Which social media platform do you think influences your aesthetic perception the most? • Instagram • TikTok • X • Facebook • Snapchat • Pinterest	(10) Do you think social media affects your aesthetic perception? • Yes • No

Data were collected between March and May 2025. A total of 242 participants were included in the study, of whom 130 were dental students, and the remaining participants were individuals outside the dental profession. All participants were selected from individuals aged 18–30 years.

**Figure 1:**
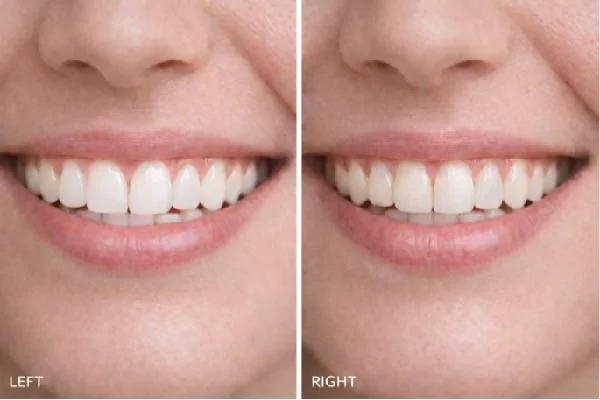
Comparison of aesthetic perception regarding tooth colour. (a) White teeth versus (b) natural tooth colour.

**Figure 2:**
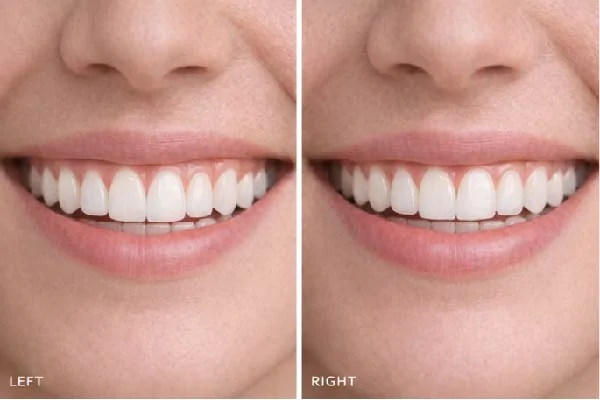
Comparison of smile aesthetics according to gingival display level. (a) High gingival display versus (b) low gingival display.

**Figure 3:**
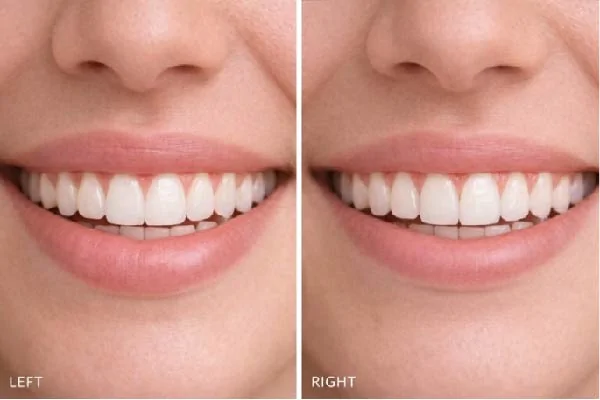
Comparison of aesthetic perception according to lip fullness. (a) Thick lips versus (b) thin lips.

**Figure 4:**
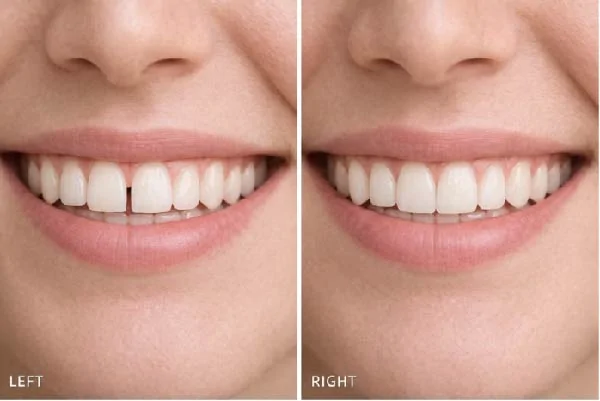
Comparison of (a) aesthetic perception between teeth with diastema and (b) well‐aligned teeth without diastema.

**Figure 5:**
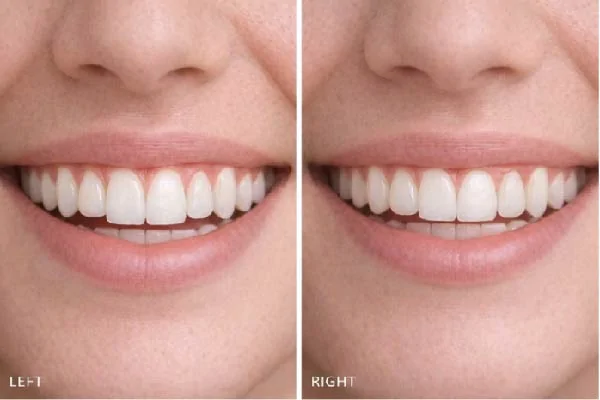
Comparison of (a) aesthetic perception between bunny teeth and (b) normal‐sized teeth.

Questions (Q) 1 aimed to obtain demographic information. Questions 2–6 consisted of visual items related to dental aesthetic perception, while questions 7–10 addressed social media usage.

The questionnaire included paired images generated using ChatGPT (OpenAI, San Francisco, CA, USA) and edited in Adobe Photoshop (Adobe Inc., San Jose, CA, USA), each evaluating specific dental aesthetic parameters. In questions 2–6, participants were asked to select the image they perceived as more aesthetic. The investigated aesthetic parameters included tooth colour (Figure 1a,b), gingival display (Figure 2a,b), lip fullness (Figure 3a,b), presence of diastema, (Figure 4a,b) ‘bunny teeth’ appearance (Figure 5a,b). Additionally, participants’ social media usage patterns, the influence of social media on dental aesthetic perception and the platforms perceived as the most influential were assessed.

The collected data were statistically analysed using R software (Version 4.4.0). Descriptive statistics were reported, and odds ratios (OR) along with *p*‐values were calculated to examine relationships between variables. The level of statistical significance was set at *α* = 0.05.

## 3. Results

‘This survey study was conducted with a total of 242 participants, of whom 130 were dental students and 112 belonged to other professional groups. Of the participants, 121 were female and 108 were male, while 13 participants preferred not to disclose their gender’.

A statistically significant difference was found between dental students and laypersons regarding gender distribution (*p* < 0.001), with laypersons predominantly male (64.4%) and dental students predominantly female (68.3%).

In Q2, the distribution of aesthetic preferences regarding tooth colour was evaluated between the groups. Both groups considered white teeth to be more aesthetic. Accordingly, no statistically significant difference was observed between the groups for this question (*p* > 0.05).

In Q3, gingival visibility was evaluated. Dental students demonstrated a higher preference for low gingival visibility compared to individuals outside the professional group. A statistically significant difference was identified between the two groups for this question, although the significance was borderline (*p* < 0.05).

In Q4, aesthetic preferences regarding lip fullness were assessed. Thin lips were more frequently preferred in both groups. Therefore, no statistically significant difference was found between the groups for this parameter (*p* > 0.05).

In Q5, the presence of diastema was evaluated in terms of the aesthetic perception between the groups. No statistically significant difference was identified (*p* > 0.05).

In Q6, the aesthetic perception of the ‘bunny teeth’ appearance was examined. Normally aligned teeth were considered more aesthetic in both groups. No statistically significant difference was observed (*p* > 0.05). The distribution of aesthetic preferences between dental students and laypersons is presented in Table 2.

**Table 2:** Comparison of aesthetic perceptions between dental students and laypersons.

Question/response options	What is your profession?		Total	Test statistic	*p*
	Laypersons (*n* = 112)	Dental students (*n* = 130)			
Q2. Aesthetic comparison of teeth with different colours					
Natural yellow teeth	51 (45.5)	48 (37.2)	99 (41.1)	1717	0.190 ^∗^
White teeth	61 (54.5)	81 (62.8)	142 (58.9)		
Q3. Comparison of gingival visibility					
Low gingival visibility	83 (74.1)	110 **(84.6)**	193 (79.8)	2913	**0.040**
High gingival visibility	29 (25.9)	20 (15.4)	49 (20.2)		
Q4. Aesthetic comparison of lip fullness					
Thick lips	72 (64.9)	75 (57.7)	147 (61)	1295	0.255 ^∗^
Thin Lips	39 (35.1)	55 (42.3)	94 (39)		

*Note:* Bold values indicate statistical significance.

^∗^Pearson chi‐square test.

Participants’ responses regarding social media usage habits and aesthetic perception were compared, and the results are presented in Table 3.

**Table 3:** Comparison of social media usage patterns and their perceived influence on aesthetic perception between dental students and laypersons.

Question/response options	What is your profession?		Total	Test statistic	*p*
	Laypersons (*n* = 112)	Dental students (*n* = 130)			
Q1. Gender					
Male	67 (64.4)	39 (31.7)	106 (46.7)	24.232	**<0.001 ^∗^**
Female	37 (35.6)	84 (68.3)	121 (53.3)		
Q7. Do you use social media?					
Yes	104 (94.6)	126 (98.4)	230 (96.6)	—	0.149 ^∗∗^
No	6 (5.5)	2 (1.6)	8 (3.4)		
Q8. Which social media platform do you use most frequently?					
Facebook	2 (1.8)	0 (0)	2 (0.8)	7.020	0.160 ^∗∗∗^
Pinterest	1 (0.9)	1 (0.8)	2 (0.8)		
Snapchat	0 (0)	1 (0.8)	1 (0.4)		
TikTok	8 (7.2)	7 (5.5)	15 (6.3)		
X	17 (15.3)	10 (7.8)	27 (11.3)		
Instagram	83 (74.8)	109 (85.2)	192 (80.3)		
Q9. Which social media platform do you think influences your aesthetic perception the most?					
Facebook	1 (0.9)	0 (0)	1 (0,4)	15.884	**0.004 ^∗∗∗^**
Pinterest	2 (1.8)^a^	10 (7.8)^b^	12 (5)		
Snapchat	0 (0)	1 (0.8)	1 (0.4)		
TikTok	12 (10.7)	6 (4.7)	18 (7.5)		
X	10 (8.9)^a^	2 (1.6)^b^	12 (5)		
Instagram	87 (77.7)	109 (85.2)	196 (81.7)		
Q10. Do you think social media affects your aesthetic perception?					
Yes	93 (83.8)	115 (88.5)	208 (86.3)	0.748	0.387 ^∗∗∗∗^
No	18 (16.2)	15 (11.5)	33 (13.7)		

*Note:* Data are presented as frequency (percentage). Bold values indicate statistical significance.

^a, b^Proportions sharing the same letter are not significantly different.

^∗^Pearson chi‐square test.

^∗∗^Fisher’s exact test.

^∗∗∗^Monte Carlo corrected Fisher’s exact test.

^∗∗∗∗^Yates’ continuity correction.

In Q7, which evaluated social media use among individuals outside the laypersons and dental students, no statistically significant difference was observed between the two groups (*p* = 0.149). A large proportion of participants reported using social media, with the majority responding ‘yes’ to the question regarding social media use (96.6%). No statistically significant difference was observed between dental students and laypersons for this variable (*p* > 0.05).

In Q8, the distribution of the most frequently used social media platform was analysed between dental students and laypersons, and no statistically significant difference was found between the groups (*p* > 0.05). Instagram was identified as the most commonly used platform in both groups (80.3%), followed by X (11.3%) and TikTok (6.3%).

However, when the social media platform perceived to have the greatest influence on aesthetic perception was examined, a statistically significant difference was detected between dental students and laypersons (Q9) (*p* < 0.05). Although Instagram ranked first in both groups, it was found to have a greater influence on the aesthetic perception of dental students (85.2%) compared to that of laypersons (77.7%).

In Q10, which assessed whether social media affects aesthetic perception, no statistically significant difference was found between dental students and laypersons (*p* > 0.05). A large proportion of participants in both groups indicated that social media influences their aesthetic perception. (88.5% in dental students and 83.8% in laypersons).

## 4. Discussion

Aesthetic perception of the smile is a complex and multifactorial concept shaped by dental characteristics, individual preferences and sociocultural influences.

The present study aimed to provide a comprehensive evaluation of dental aesthetic perception by assessing multiple aesthetic parameters and the potential influence of social media. Understanding these factors is essential for improving patient‐centred treatment planning and aligning professional evaluations with patient expectations.

Previous studies have reported differences in the perception of smile aesthetics among dental professionals, dental students and laypersons [11, 15–18].

Among the various dental aesthetic parameters, the gingival display is one of the most influential factors in smile attractiveness. Several studies have indicated that minimal gingival display during smiling is generally perceived as more attractive [19–21]. However, Suzuki et al. [22] reported that gingival display is not necessarily considered an unfavourable aesthetic feature. In the present study, images with reduced gingival display were preferred as more aesthetic in both groups, with a statistically significant difference between dental students and laypersons. Although the majority of laypersons also preferred minimal gingival display, a relatively higher proportion selected images with increased gingival visibility compared to that of dental students. This difference may be attributed to the fact that the study conducted by Suzuki et al. [22] included only laypersons, whereas the present study involved both dental students and laypersons. Furthermore, while the majority of laypersons in the present study preferred reduced gingival display, the proportion of participants selecting increased gingival display was higher compared to that of dental students. This finding suggests that gingival display may influence the aesthetic perception regardless of the evaluator’s level of knowledge and experience, although this effect appears to be more pronounced among dental students due to their familiarity with established aesthetic norms. In contrast, the relatively higher tolerance observed among laypersons may reflect differences in the aesthetic perception and acceptance thresholds between the groups.

Another important dental aesthetic parameter is the presence of diastema, which has been reported to negatively affect perceived smile attractiveness in several studies [16, 23, 24]. In a study evaluating different dental aesthetic parameters and involving a sample group similar to the present study, diastema was found to have the lowest aesthetic rating [10]. Similarly, another study reported that all participants evaluated the presence of diastema as less aesthetic [24]. Consistent with these findings, the diastema was rated as less aesthetic by both groups in the present study.

In addition to structural features, tooth colour is a key determinant of dental aesthetics [25]. Previous studies have shown that younger individuals tend to prefer lighter tooth shades [11, 26]. Vallittu et al. [27] reported that younger individuals prefer lighter tooth tones compared to older individuals. In the present study, participants were within a young age range, and a lighter tooth colour was preferred as more aesthetic. In a study involving a similar age group, participants reported dissatisfaction with their natural tooth colour and expressed a preference for whiter teeth [28].

Regarding facial soft tissue components, lip fullness is also an important factor influencing smile aesthetics. In the present study, thicker lips were perceived as more aesthetic than thinner lips in both groups, which is consistent with several studies [29, 30]. Kar et al. [31] reported that fuller lips tend to be perceived as more attractive, particularly in females. However, in another study, Sadrhaghighi et al. [32] stated that changes in lip thickness were not distinctly perceived. These differences may be attributed to methodological variations across studies.

In recent years, emerging aesthetic trends such as the ‘bunny teeth’ appearance have gained attention, particularly through social media. In the present study, this appearance was compared with normal tooth alignment, and the majority of participants (93.8%) preferred normal tooth alignment as being more aesthetic. This finding suggests that despite the increasing visibility of such trends, traditional aesthetic norms remain dominant.

Beyond individual dental parameters, external factors such as social media have also emerged as important determinants of the aesthetic perception. Social media platforms, due to their accessibility, continuously updated content and widespread global use, have become powerful tools for shaping individuals’ perceptions and attitudes [33]. Previous studies have reported that commonly used platforms such as Facebook, Instagram, X and Snapchat play a significant role in influencing the aesthetic perception [33]. Murray et al. [34] demonstrated that even brief exposure to advertisements can influence individuals’ aesthetic perception. In the present study, 83.8% of laypersons and 88.5% of dental students reported that social media influences their aesthetic perception, which is consistent with the findings of Abbasi et al. [35], who reported that social media affects individuals’ satisfaction with their dental appearance. Additionally, Instagram was identified as the most frequently used social media platform (80.3%), which is also consistent with the findings of Abbasi et al. [35] However, in contrast, Bahabri and Zaidan [36] and Parmar et al. [37] reported Facebook as the most commonly used platform. These differences may be attributed to variations in age distribution and regional differences in social media usage patterns.

The increasing use of social media in daily life may play a significant role in shaping individuals’ perceptions of dental aesthetics. The prominence of Instagram as the most frequently used platform and as the platform with the greatest influence on aesthetic perception (81.7%) highlights the potential impact of visually oriented platforms on the aesthetic judgment. Furthermore, the high proportion of participants reporting that social media influences their aesthetic perception supports findings from Atabaki and Duijster [38], who suggested that social media may indirectly influence aesthetic treatment decisions by promoting certain beauty standards.

The high level of perceived influence of social media among dental students also highlights the importance of incorporating social media literacy into dental education. This would enable future professionals to critically evaluate digitally driven and sometimes unrealistic aesthetic expectations [39, 40]. Overall, the findings of this study suggest that dental students and laypersons demonstrated largely similar preferences regarding dental aesthetic parameters, with no pronounced differences between the groups. In contrast, the social media were found to have a substantial influence on the aesthetic perception in both groups. The high proportion of participants reporting this influence highlights the growing role of digital platforms in shaping contemporary aesthetic standards. These findings emphasise the importance of considering the impact of social media, in addition to clinical criteria, when evaluating patient expectations in an aesthetic dental practice.

This study has several limitations. The unequal gender distribution between dental students and laypersons may have influenced the findings as potential gender‐related differences in aesthetic perception could not be fully controlled. In addition, the single‐centre design may limit the generalisability of the results. The use of digitally generated and edited images may not fully reflect real‐life clinical conditions. Moreover, the aesthetic perception was evaluated using a limited number of parameters, and other influencing factors were not included. The cross‐sectional nature of the study also prevents the assessment of changes in the aesthetic perception over time.

## 5. Conclusion

Within the limitations of this study, it can be concluded that dental students and laypersons showed largely similar preferences regarding dental aesthetic parameters, with no consistent or pronounced differences between the groups.

However, the social media was found to have a substantial influence on the aesthetic perception in both groups. A high proportion of participants reported that their aesthetic preferences were affected by social media, highlighting its growing role in shaping contemporary perceptions of dental aesthetics.

These findings emphasise the importance of considering the impact of social media alongside clinical criteria when evaluating patient expectations in the aesthetic dental practice.

## Funding

This research did not receive any specific grant from funding agencies in the public, commercial or not‐for‐profit sectors.

## Consent

The authors have nothing to report.

## Conflicts of Interest

The authors declare no conflicts of interest.

## Data Availability

The data that support the findings of this study are available upon request from the corresponding author. The data are not publicly available due to privacy or ethical restrictions.

## References

[1] El Mourad A. M. , Al Shamrani A. , Al Mohaimeed M. , Al Sougi S. , Al Ghanem S. , and Al Manie W. , Self-Perception of Dental Esthetics Among Dental Students at King Saud University and Their Desired Treatment, International Journal of Dentistry. (2021) 2021, no. 1, 10.1155/2021/6671112, 6671112. PMC7925033 33679980

[2] Ioi H. , Kang S. , and Shimomura T. , et al.Effects of Buccal Corridors on Smile Esthetics in Japanese and Korean Orthodontists and Orthodontic Patients, American Journal of Orthodontics and Dentofacial Orthopedics. (2012) 142, no. 4, 459–465, 10.1016/j.ajodo.2012.05.011. 22999668

[3] Sarver D. M. and Ackerman M. B. , Dynamic Smile Visualization and Quantification: Part 2. Smile Analysis and Treatment Strategies, American Journal of Orthodontics and Dentofacial Orthopedics. (2003) 124, no. 2, 116–127, 10.1016/S0889-5406(03)00307-X. 12923505

[4] Armalaite J. , Jarutiene M. , and Vasiliauskas A. , et al.Smile Aesthetics as Perceived by Dental Students: A Cross-Sectional Study, BMC Oral Health. (2018) 18, no. 1, 10.1186/s12903-018-0673-5. PMC6303883 30577772

[5] Aldhorae K. , Alqadasi B. , Altawili Z. M. , Assiry A. , Shamalah A. , and Addin Al-Haidari S. , Perception of Dental Students and Laypersons to Altered Dentofacial Aesthetics, Journal of International Society of Preventive and Community Dentistry. (2020) 10, no. 1, 85–95, 10.4103/jispcd.JISPCD_340_19. PMC7055345 32181225

[6] Cheng J. H. C. , Hsu Y. C. , Lee T. Y. H. , and Li R. W. , Factors Affecting Perception of Laypeople and Dental Professionals Toward Different Smile Esthetics, Journal of Dental Sciences. (2023) 18, no. 2, 739–746, 10.1016/j.jds.2022.11.030. PMC10068377 37021227

[7] Pithon M. M. , Santos A. M. , de Andrade A. C. D. V. , Santos E. M. , Couto F. S. , and da Silva Coqueiro R. , Perception of the Esthetic Impact of Gingival Smile on Laypersons, Dental Professionals, and Dental Students, Oral Surgery, Oral Medicine, Oral Pathology and Oral Radiology. (2013) 115, no. 4, 448–454, 10.1016/j.oooo.2012.04.027. 23022025

[8] Althagafi N. , Esthetic Smile Perception Among Dental Students at Different Educational Levels, Clinical, Cosmetic and Investigational Dentistry. (2021) 13, 163–172, 10.2147/CCIDE.S304216. PMC8112856 33994813

[9] Amin E. , Khan M. , Siddiqui M. O. , Babar A. , Hassan F. , and Ejaz W. , Perception of Altered Smile Esthetic Among Dentists, Students and Laypersons Using Digital Photographs: A Comparative Study, Pakistan Armed Forces Medical Journal. (2021) 71, no. 3, 10.51253/pafmj.v71i3.4592.

[10] Cracel-Nogueira F. and Pinho T. , Assessment of the Perception of Smile Esthetics by Laypersons, Dental Students and Dental Practitioners, International Orthodontics. (2013) 11, no. 4, 432–444, 10.1016/j.ortho.2013.09.007. 24427802

[11] Alharthi A. K. , Mohamed R. N. , Bailoor D. N. , Bassuoni M. W. , Shaikh W. A. , and Al-Thomali Y. , Perception of Smile and Esthetic Among Dental and Non-Dental Student, Annals of International medical and Dental Research. (2018) 4, no. 2, 59–63, 10.21276/aimdr.2018.4.2.DE13.

[12] Sipiyaruk K. , Chanvitan P. , Kongton N. , Runggeratigul P. , and Niyomsujarit N. , The Impact of Smile Appearance and Self-Perceived Smile Attrac Tiveness on Psychological Well-Being Amongst Dental Undergraduates, International Journal of Clinical Dentistry. (2022) 15, no. 2, 365–378.

[13] Zaidi A. B. , Karim A. A. , Mohiuddin S. , and Rehman K. , Effects of Dental Aesthetics on Psycho-Social Wellbeing Among Students of Health Sciences, Journal of the Pakistan Medical Association. (2020) 70, no. 6, 1002–1005. 32810096

[14] Baik K. M. , Anbar G. , Alshaikh A. , and Banjar A. , Effect of Social Media on Patient’s Perception of Dental Aesthetics in Saudi Arabia, International Journal of Dentistry. (2022) 2022, no. 1, 10.1155/2022/4794497, 4794497. PMC8898879 35265132

[15] Torul D. and Omezli M. M. , The Influence of Education and Gender on the Esthetic Perception of Facial Profile, The International Journal of Esthetic Dentistry. (2022) 17, no. 1, 88–99. 35175010

[16] Talic N. , AlOmar S. , and AlMaidhan A. , Perception of Saudi Dentists and Lay People to Altered Smile Esthetics, The Saudi Dental Journal. (2013) 25, no. 1, 13–21, 10.1016/j.sdentj.2012.09.001. PMC3723121 23960550

[17] Ren H. , Chen X. , and Zhang Y. , Correlation Between Facial Attractiveness and Facial Components Assessed by Laypersons and Orthodontists, Journal of Dental Sciences. (2021) 16, no. 1, 431–436, 10.1016/j.jds.2020.07.012. PMC7770325 33384831

[18] Geevarghese A. , Baskaradoss J. K. , and Alsalem M. , et al.Perception of General Dentists and Laypersons Towards Altered Smile Aesthetics, Journal of Orthodontic Science. (2019) 8, no. 1, 10.4103/jos.JOS_103_18. PMC6702677 31497573

[19] Zawawi K. , Malki G. A. , Al-Zahrani M. S. , and Alkhiary Y. M. , Effect of Lip Position and Gingival Display on Smile and Esthetics as Perceived by College Students With Different Educational Backgrounds, Clinical, Cosmetic and Investigational Dentistry. (2013) 10.2147/CCIDE.S53359. PMC3816985 24204173

[20] Al Taki A. , Hayder Mohammed T. , and Mohammad Hamdan A. , Influence of the Smile Line on Smile Attractiveness in Short and Long Face Individuals, International Journal of Dentistry. (2017) 2017, 10.1155/2017/2637148, 2637148. PMC5591915 28928770

[21] Machado A. W. , 10 Commandments of Smile Esthetics, Dental Press Journal of Orthodontics. (2014) 19, no. 4, 136–157, 10.1590/2176-9451.19.4.136-157.sar. PMC4296640 25279532

[22] Suzuki L. , Machado A. W. , and Bittencourt M. A. V. , An Evaluation of the Influence of Gingival Display Level in the Smile Aesthetics, Dental Press Journal of Orthodontics. (2011) 16, no. 5, 1–10, 10.1590/S2176-94512011000500005.

[23] Aldegheishem A. , Alfayadh H. M. , and AlDossary M. , et al.Perception of Dental Appearance and Aesthetic Analysis Among Patients, Laypersons and Dentists, World Journal of Clinical Cases. (2024) 12, no. 23, 5354–5365, 10.12998/wjcc.v12.i23.5354. PMC11238684 39156080

[24] Rabe A. H. , Tabchi Y. , Zaoui F. , Faouzi A. M. , and Bahoum A. , Perception of Smile Aesthetics Between Orthodontists and Moroccan Patients, Indian Journal of Dental Research. (2023) 34, no. 2, 178–181, 10.4103/ijdr.ijdr_179_22. 37787208

[25] Joiner A. and Luo W. , Tooth Colour and Whiteness: A Review, Journal of Dentistry. (2017) 67S, S3–S10, 10.1016/j.jdent.2017.09.006. 28928097

[26] Pavicic D. K. , Spalj S. , Uhac I. , and Lajnert V. , A Cross-Sectional Study of the Influence of Tooth Color Elements on Satisfaction With Smile Esthetics, The International Journal of Prosthodontics. (2017) 30, no. 2, 156–159, 10.11607/ijp.5070. 28267826

[27] Vallittu P. K. , Vallittu A. S. J. , and Lassila V. P. , Dental Aesthetics—A Survey of Attitudes in Different Groups of Patients, Journal of Dentistry. (1996) 24, no. 5, 335–338, 10.1016/0300-5712(95)00079-8. 8916647

[28] Isiekwe G. I. and Aikins E. A. , Self-Perception of Dental Appearance and Aesthetics in a Student Population, International Orthodontics. (2019) 17, no. 3, 506–512, 10.1016/j.ortho.2019.06.010. 31255551

[29] Rai D. , Janardhanam P. , and Rai A. , Esthetic Factors of Smile in Vertical Dimensions: A Comparative Evaluation, Journal of Indian Orthodontic Society. (2015) 49, no. 1, 25–31, 10.4103/0301-5742.158630.

[30] Krajangta N. , Phumpatrakom P. , Supachartwong C. , Kamolwarin P. , and Leelaponglit S. , Influence of Lip Appearances and Tooth Shade on Smile Attractiveness Perception, International Journal of Dentistry. (2022) 2022, no. 1, 10.1155/2022/5952643, 5952643. PMC9788899 36571069

[31] Kar M. , Muluk N. B. , Bafaqeeh S. A. , and Cingi C. , Is it Possible to Define the Ideal Lips?, Acta Otorhinolaryngologica Italica. (2018) 38, no. 1, 67–72, 10.14639/0392-100X-1511. PMC5952987 29756617

[32] Sadrhaghighi H. , Zarghami A. , Sadrhaghighi S. , and Eskandarinezhad M. , Esthetic Perception of Smile Components by Orthodontists, General Dentists, Dental Students, Artists, and Laypersons, Journal of Investigative and Clinical Dentistry. (2017) 8, no. 4, 10.1111/jicd.12235, e12235. 27590143

[33] Arab K. , Barasain O. , and Altaweel A. , et al.Influence of Social Media on the Decision to Undergo a Cosmetic Procedure, Plastic and Reconstructive Surgery-Global Open. (2019) 7, no. 8, 10.1097/GOX.0000000000002333, e2333. PMC6756652 31592374

[34] Murray S. H. , Touyz S. W. , and Beumont P. J. V. , Awareness and Perceived Influence of Body Ideals in the Media: A Comparison of Eating Disorder Patients and the General Community, Eating Disorders. (1996) 4, no. 1, 33–46, 10.1080/10640269608250074.

[35] Abbasi M. S. , Lal A. , and Das G. , et al.Impact of Social Media on Aesthetic Dentistry: General Practitioners’ Perspectives, Healthcare. (2022) 10, no. 10, 10.3390/healthcare10102055, 2055. PMC9602612 36292502

[36] Bahabri R. H. and Zaidan A. B. , The Impact of Social Media on Dental Practice Promotion and Professionalism Amongst General Dental Practitioners and Specialists in KSA, Journal of Taibah University Medical Sciences. (2021) 16, no. 3, 456–460, 10.1016/j.jtumed.2020.12.017. PMC8178643 34140874

[37] Parmar N. , Dong L. , and Eisingerich A. B. , Connecting With Your Dentist on Facebook: Patients’ and Dentists’ Attitudes Towards Social Media Usage in Dentistry, Journal of Medical Internet Research. (2018) 20, no. 6, 10.2196/10109, e10109. PMC6045786 29959108

[38] Atabaki A. and Duijster D. , The Role of Social Media in Patient Decision-Making in Choosing Dental Bleaching Treatments, Community Dental Health. (2025) 42, no. 4, 102–111, 10.1177/0265539X251361703. 40670156

[39] Özişçi Ö. , Gökoğlu S. S. , and Ay B. , Perceptions and Predictors of Dental Esthetic Satisfaction Among Turkish Dental Students: The Role of Social and Professional Factors, BMC Medical Education. (2025) 26, no. 1, 10.1186/s12909-025-08414-x. PMC12801982 41353152

[40] Dahong X. , Xiangrong C. , Ying L. , Yusong L. , Ying G. , and Yan S. , Effect of Incisor Position on the Self-Perceived Psychosocial Impacts of Malocclusion Among Chinese Young Adults, The Angle Orthodontist. (2013) 83, no. 4, 617–622, 10.2319/062012-508.1. PMC8754026 23163832

